# Genome-Wide Identification and Transcriptomic Analysis of *MYB* Transcription Factors in Seashore Paspalum Under Salt Stress

**DOI:** 10.3390/ijms27021068

**Published:** 2026-01-21

**Authors:** Yuzhu Wang, Xuanyang Wu, Qi Sun, Wenjie Lu, Zhanfeng Ren, Zeng-Yu Wang, Xueli Wu

**Affiliations:** 1 College of Grassland Science, Qingdao Agricultural University, Qingdao 266000, China; 2 Shandong Key Laboratory for Germplasm Innovation of Saline-Alkaline Tolerant Grasses and Trees, Qingdao 266000, China

**Keywords:** seashore paspalum, *PvMYBs*, gene family, salt stress

## Abstract

The MYB transcription factor family plays crucial roles in plant growth, development, and responses to biotic and abiotic stresses. Seashore paspalum (*Paspalum vaginatum*) is a halophytic grass species with remarkable salt tolerance, yet its *MYB* gene family has not been systematically characterized. In this study, we conducted a genome-wide identification of *MYB* genes in seashore paspalum using a Hidden Markov Model (HMM)-based approach, resulting in the identification of 157 *PvMYB* genes. Phylogenetic and conserved motif analyses revealed distinct subfamily groupings and evolutionary relationships within the PvMYB family. Promoter analysis indicated that *PvMYB* genes contain multiple cis-acting elements responsive to light, hormones, and abiotic stresses, suggesting their potential regulatory roles under salt stress. Collinearity and duplication analyses demonstrated that gene duplication events contributed to the expansion of the PvMYB family. Moreover, protein interaction network prediction suggested that *PvMYB73* may interact with key regulatory proteins such as BZIP8 and DREB1F involved in salt stress signaling. Transcriptome and reverse transcription quantitative PCR (RT-qPCR) analyses showed that *PvMYB90*, *PvMYB123*, and *PvMYB150* were upregulated in leaves and roots under salinity stress, while *PvMYB85* and *PvMYB90* were experimentally validated to localize in the nucleus and function in salt tolerance regulation. Collectively, this study provides the first comprehensive characterization of the *MYB* gene family in seashore paspalum and offers valuable insights into the molecular mechanisms underlying salt tolerance in halophytic grasses.

## 1. Introduction

Plants are constantly challenged by a wide array of environmental stresses throughout their growth and development, which can be broadly divided into biotic and abiotic stresses. Biotic stresses mainly arise from pathogenic infections, insect herbivory, and weed competition, whereas abiotic stresses encompass drought, flooding, salinity, extreme temperatures, excessive or insufficient light, heavy metal toxicity, and nutrient imbalances [1,2]. These adverse factors impose intricate effects on plant morphology, physiology, and molecular metabolism, often resulting in stunted growth, metabolic disturbances [3,4], and reduced yield and quality [5].

Among these abiotic stresses, salt stress is one of the most pervasive and detrimental constraints on global agricultural productivity. Presently, approximately 40% of global arable land suffers from varying degrees of salinity stress, with deteriorating irrigation water quality exacerbating the accumulation of soil salts and alkaline substances, thereby suppressing plant growth and threatening food security [6,7,8]. Salt stress primarily disrupts cellular ion homeostasis and osmotic balance, leading to water deficit, oxidative stress, and impaired metabolic function [9,10]. High salinity triggers excessive accumulation of sodium ions (Na^+^), which interferes with potassium (K^+^) uptake, disturbs enzymatic activities, and damages membrane integrity [11]. Consequently, plants exposed to salinity exhibit inhibited photosynthesis, reduced growth, and premature senescence [12].

To cope with environmental adversities, plants have evolved sophisticated adaptive mechanisms, including osmotic adjustment, ion compartmentalization, activation of antioxidant defense systems, and transcriptional reprogramming of stress-responsive genes [13]. Among these, transcription factors (TFs) play pivotal roles in coordinating stress signaling networks by modulating the expression of downstream genes involved in osmolyte biosynthesis, reactive oxygen species (ROS) detoxification, and hormonal signaling pathways [14]. For instance, overexpression of *McWRKY57*-like in transgenic Arabidopsis significantly enhances drought tolerance by regulating plant growth, osmolyte accumulation, antioxidant enzyme activities, and the transcription of stress-related genes [15]. In rice, phosphorylation of *OsERF52* by *OsSAPK9* strengthens its interaction with IPA1 and *OsbHLH002*, thereby inducing *OsCBF* gene expression and conferring improved chilling tolerance [16]. Likewise, overexpression of the wheat WRKY transcription factor TaWRKY17 markedly enhances salt tolerance in both transgenic Arabidopsis and wheat by activating antioxidant defense pathways and stress-responsive genes [17]. Moreover, the rice MADS-box transcription factor *OsMADS57* has been reported to enhance salt tolerance through modulation of antioxidant enzyme activity and regulation of multiple stress-responsive signaling cascades [18]. Collectively, these studies demonstrate that, mining key genes conferring saline-alkaline tolerance, in combination with molecular design breeding strategies, enables the development of novel crop cultivars to overcome grain yield limitations in saline-alkaline soils.

Seashore paspalum (*Paspalum vaginatum* O. Swartz, 2n = 2x = 20), is a halophytic grass species in the Poaceae family, phylogenetically related to major cereals such as rice (*Oryza sativa*) and maize (*Zea mays*). It is naturally distributed in tropical and subtropical regions, particularly along coastal areas and sandy soils where high salinity conditions prevail [19]. It is recognized for its vigorous growth, dense turf formation, and exceptional tolerance to salinity, making it widely used for coastal landscaping, golf courses, sports fields, and as forage grass. Remarkably, its salt tolerance is nearly twice that of Bermudagrass (*Cynodon dactylon*), allowing it to thrive under irrigation with saline or even seawater conditions [20]. This outstanding tolerance, together with its strong regenerative and adaptive capacity, renders seashore paspalum a promising candidate for ecological restoration and the reclamation of saline–alkaline soils [21]. As a halophyte with high salinity resilience, seashore paspalum provides an excellent system for studying molecular mechanisms of salt tolerance in grasses. Identifying and characterizing key salt-responsive genes from this species may yield valuable genetic resources for the molecular breeding of stress-resilient cereal crops.

MYB transcription factors constitute the largest and most functionally diverse transcription factor family in plants [22,23], and they play pivotal roles in regulating plant growth/development and mediating responses to abiotic stresses including salt stress [24,25,26,27]. Their structural architecture is characterized by 1–4 conserved MYB repeats (R), with each R domain comprising approximately 50–53 amino acid residues that form three α-helices and bind to the major groove of target gene DNA via a helix-turn-helix (HTH) motif [28]. Plant MYB transcription factors are classified into four classes based on MYB repeat number: 1R-MYB (MYB-like), 2R-MYB (R2R3-MYB), 3R-MYB (R1R2R3-MYB), and 4R-MYB [29], among which the R2R3-MYB subfamily is the most abundant and most extensively studied [30,31]. MYB transcription factors function as critical regulators in plant responses to saline-alkaline stress. For instance, overexpression of tomato (*Solanum lycopersicum*) *SlMYB102* significantly enhanced salt tolerance in transgenic plants [32,33]. Similarly, overexpression of the grape (*Vitis vinifera* L.) transcription factor *VhMYB2* enhances salt tolerance in *Arabidopsis thaliana* [34], and overexpression of strawberry (*Fragaria vesca)* transcription factor *FvMYB44* enhances salt tolerance in *Arabidopsis thaliana* [35]. In upland cotton (*Gossypium hirsutum*), elevated expression of *GhMYB73* conferred superior salt tolerance [36]. Furthermore, overexpression of soybean (*Glycine max*) *GmMYB68* not only enhanced salt tolerance but also improved pod yield under saline conditions [37]. These studies collectively demonstrate the indispensable regulatory role of MYB transcription factors in plant responses and adaptation to salt stress.

Although the critical regulatory role of MYB transcription factors in plant salt stress response has been extensively demonstrated across various crop species, their functional mechanisms in halophytic grasses such as seashore paspalum remain largely unexplored. In this study, we conducted genome-wide identification and bioinformatic analysis of the *MYB* gene family in seashore paspalum, elucidating its repertoire, conserved domains, promoter cis-regulatory elements, and phylogenetic relationships. Based on transcriptomic data, we further identified and cloned two key salt stress-responsive genes, *MYB85* and *MYB90*, thereby providing valuable genetic resources for deciphering the molecular mechanisms underlying salt tolerance in seashore paspalum and for the genetic improvement of stress resilience in cereal and forage crops.

## 2. Results

### 2.1. Identification and Physicochemical Property Analysis of the MYB Gene Family in Seashore Paspalum

**Figure 1:**
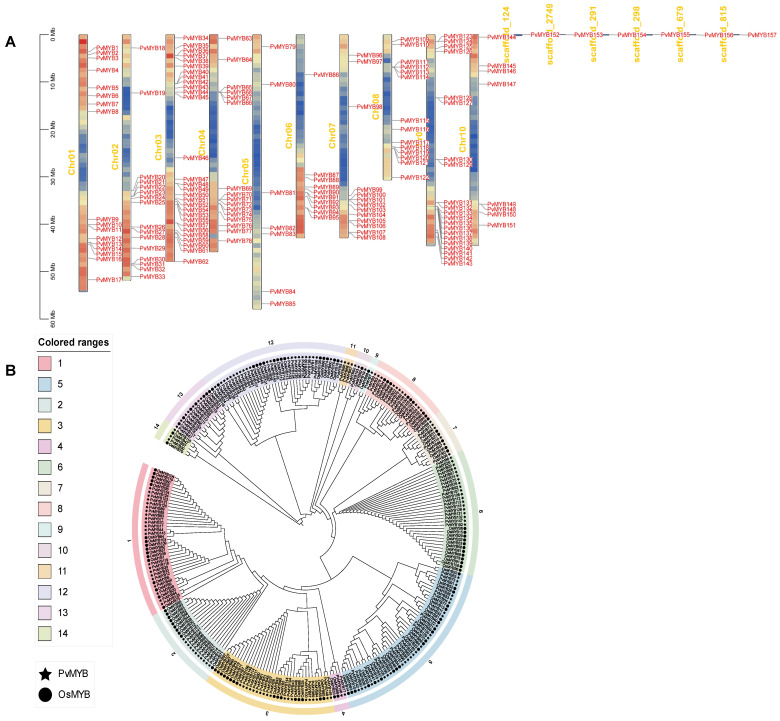
Chromosomal distribution and phylogenetic analysis of *MYB* genes in seashore paspalum. (**A**) The figure illustrates the distribution of *PvMYB* genes across chromosomes and scaffolds in seashore paspalum. All named *PvMYB* genes are displayed on the chromosomes of seashore paspalum, with chromosome numbers labeled at the top of each bar. The lines within each chromosome indicate gene density. (**B**) Maximum likelihood (ML) phylogenetic tree constructed using MEGA 11 (v0.13) based on full-length PvMYB and rice OsMYB protein sequences. The tree was divided into 14 distinct clades (color-coded). Black stars denote PvMYBs; black circles denote OsMYBs. Numbers at nodes indicate bootstrap support values (>70%) from 1000 replicates.

Using Hidden Markov Model (HMM) [38] for genome-wide screening, we identified 157 MYB transcription factors in seashore paspalum. These loci were redesignated as PvMYB1–PvMYB157 according to their positions along the chromosomes. PvMYBs were non-uniformly distributed across the 10 chromosomes of this species. To visualize the chromosomal distribution of *PvMYB* genes, chromosomal location data from the seashore paspalum genome annotation file were used to generate a PvMYB chromosomal localization map (Figure 1A). The 157 PvMYB transcription factors display a non-uniform and irregular dispersal throughout the 10 chromosomes (Chr01–Chr10) in seashore paspalum. The majority of PvMYBs transcription factors are located on Chr03 (29 genes) and Chr09 (21 genes), whereas fewer are found on Chr05 (7 genes) and Chr10 (8 genes). Additionally, six transcription factors (*PvMYB152*, *PvMYB153*, *PvMYB154*, *PvMYB155*, *PvMYB156*, and *PvMYB157*) remain unmapped to any chromosome, potentially due to limitations in genome assembly.

To investigate the evolutionary relationships of MYB proteins in seashore paspalum, phylogenetic relationships were constructed using the MEGA 11(v0.13) software [39] (Figure 1B). The phylogenetic tree was constructed based on full-length amino acid sequences of 117 MYB transcription factors from rice and 157 predicted MYB transcription factors from seashore paspalum. The PvMYB was classified into 14 distinct groups with one, Clade 5, with the largest number, 47 proteins, and one, Clade 9, with the smallest number, 2. This distribution appears to indicate strong phylogenetic relationships in this family of MYB transcription factors.

For the proteins translated from these 157 PvMYBs, predictions encompassed core attributes such as amino acid chain length, approximate molecular mass, isoelectric point, and pH profile (Supplementary Table S1). Subcellular localization analysis using Wolf PSORT [40] further demonstrated that, with the exception of PvMYB47, all remaining 156 proteins were predicted to localize to the nucleus, consistent with their functional characteristics as transcription factors (Supplementary Table S2). PvMYB47 is the shortest (78 aa); subsequent domain analysis suggests it contains only a partial MYB motif. Given its limited length, PvMYB47 likely represents a pseudogene or a truncated MYB-like protein, rather than a canonical functional R2R3-MYB.

### 2.2. Conserved Motif Analysis and Domain Visualization of the MYB Gene Family in Seashore Paspalum

**Figure 2:**
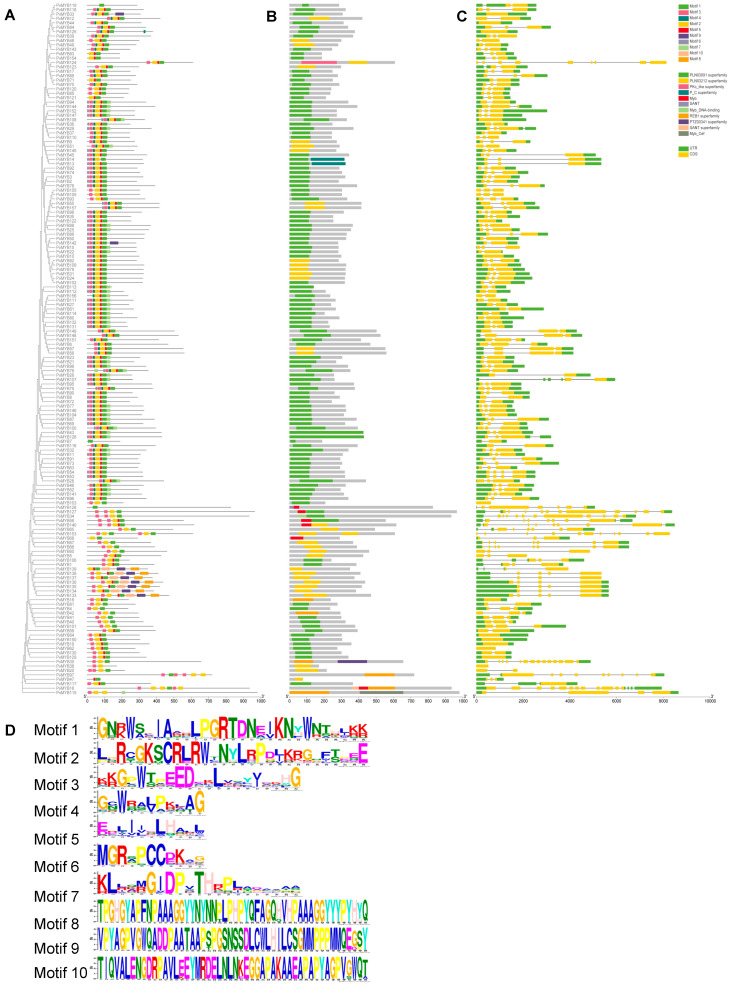
Phylogenetic relationships, conserved motifs, domain architectures, and gene structures of 157 *PvMYB* genes in seashore paspalum. (**A**) Maximum likelihood (ML) phylogenetic tree of *PvMYB* genes in seashore paspalum, showing the positions of 10 conserved motifs across the proteins, with different colors indicating different motifs. (**B**) Domain architecture of PvMYB proteins. (**C**) The gene structure of *PvMYB* genes, where yellow boxes, green boxes, and gray lines represent exons, untranslated regions (UTRs), and introns, respectively. The scale at the bottom is used to estimate their lengths. (**D**) Black, hydrophobic amino acid; green, polar amino acid; blue, positively charged amino acid; red, negatively charged amino acid; purple, neutral amino acid.

The MEME web [41] tool facilitated examination of conserved motifs across the PvMYB protein sequences (Figure 2B), while TBtools (v1.108) [42] enabled visualization of these findings integrated with the phylogenetic tree. Overall, ten distinct motifs were identified, varying in size from 11 to 50 residues (Figure 2A,D). Among the 157 members of the MYB family, motif 2 has the highest occurrence frequency, followed by motif 5. The number of motifs varies among individual members of the MYB family, that is, some members of the MYB family contain 1–7 motifs. In the phylogeny the proteins from closely related subfamilies are more conserved and show similar presence of the motifs. This may be some reason for the functional differentiation among the genes L. Each of the *PvMYB* genes studied contain the signature conserved region of the MYB family which is related to the N-terminal end of the polypeptide chain; however, a little more than 10 per cent of the regions of the motifs are in the central or C-terminal regions of the polypeptide. The location of the motifs indicates their very important role in the manifestation of the biological activities of MYB proteins. Overall, the proteins in the phylogenetic clusters are more similar in regard to the presence of motifs, which again indicates that the homologs of the different members of clusters probably have similar biological functions. In order to obtain information relative to the architecture of the genes of the MYB family, we examined the exon–intron structure and made a schematic representation of all 157 loci of the MYB family (Figure 2C). Most of the *PvMYB* genes contain just three exons. The various members of the subfamilies likewise have exons of a similar size, in a similar place and varying in number in a similar way. This fact points strongly to a high degree of evolutionary conservation of the structural aspect of the family of *MYB* genes. Collectively, the motif composition and exon–intron structure of the *PvMYB* gene family are highly consistent with the phylogenetic relationships, providing a clear structural basis and evolutionary evidence for their functional similarity and divergence.

### 2.3. Analysis of Cis-Acting Elements in the Promoter Regions of the MYB Gene Family in Seashore Paspalum

**Figure 3:**
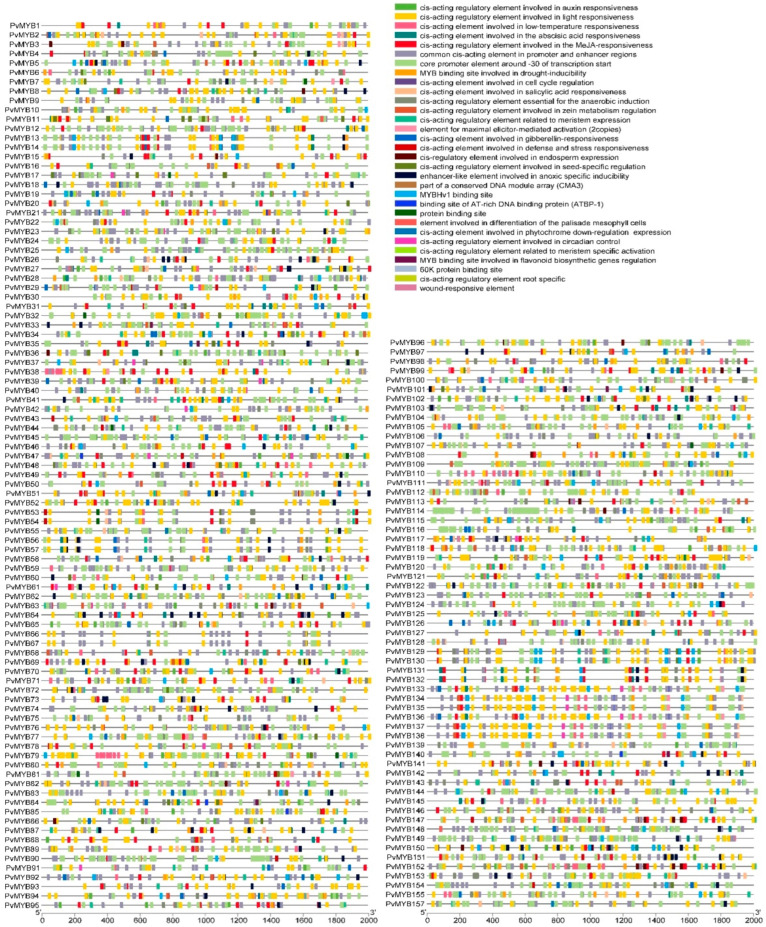
Cis-acting element analysis of *MYB* transcription factors in seashore paspalum. The figure illustrates the distribution of cis-regulatory elements in the promoter regions of PvMYB transcription factors. Different colors represent various cis-elements associated with responses to environmental stresses and hormones.

To dissect the transcriptional regulatory mechanisms of *PvMYB* transcription factors in hormone signaling and stress responses, cis-element prediction analysis was performed on the 2000 bp promoter regions upstream to systematically identify core regulatory sequences associated with hormone, biotic, and abiotic stress responses (Supplementary Table S3). The *MYB* gene family in this species has a number of these cis-regulatory elements concerned with hormonal interactions and ecological systems (Figure 3). The elaborate regulatory mechanism of this family contains elements, which are responsive to gibberellins, abscisic acid, drought stress, auxins, cold stress, protein catabolism, as well as myb-binding sites. In summary, this information indicates that the *PvMYB* family of genes is involved in hormonal function alteration, environmental stress resistance, photoperception, etc. The patterns here observed also parallel those already discussed in earlier investigations of the MYB functions.

### 2.4. Collinearity and Evolution Analysis of PvMYBs

**Figure 4:**
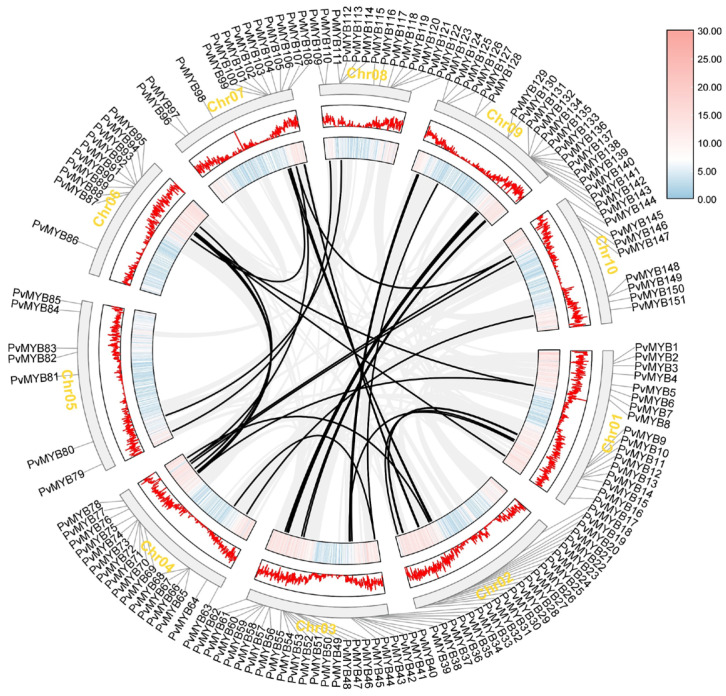
Intraspecific collinearity analysis of the *MYB* gene family in seashore paspalum. Gray boxes represent chromosomes, black lines indicate *PvMYB* gene pairs, and gray lines represent other homologous gene pairs in seashore paspalum. The black lines highlight duplicated gene pairs, suggesting potential evolutionary events within the *MYB* gene family.

To assess the evolution of the *PvMYB* gene families, we performed an analysis of the synteny analysis of the PvMYB loci, also at the intraspecific but at the synteny level. Duplication events are important components of the evolution of plant speciation where copies of tandem, dispersed, or segmental origin affect significantly the pattern of the multigenic families or clusters as well as the complexity of the chromosomal structure. The results showed that 157 genes of the *PvMYB* were mapped by the chromosomal location of the genome of the seashore paspalum with the black line indicating the occurrence of duplications. The *PvMYB* was the gene family that had 40 collinear gene pairs of which the chromosome 4 was the chromosome that presented the greater number of duplicated genes. The gene duplication events are distributed across all 10 chromosomes, and the distribution is uneven (Figure 4). These observations underscore the role of gene duplications in driving the evolutionary expansion of the PvMYB family, likely facilitating functional diversification and adaptive traits such as enhanced stress tolerance in this halophytic species.

### 2.5. Transcriptomic and Differential Gene Expression Analysis of Seashore Paspalum Under Salt Stress

**Figure 5:**
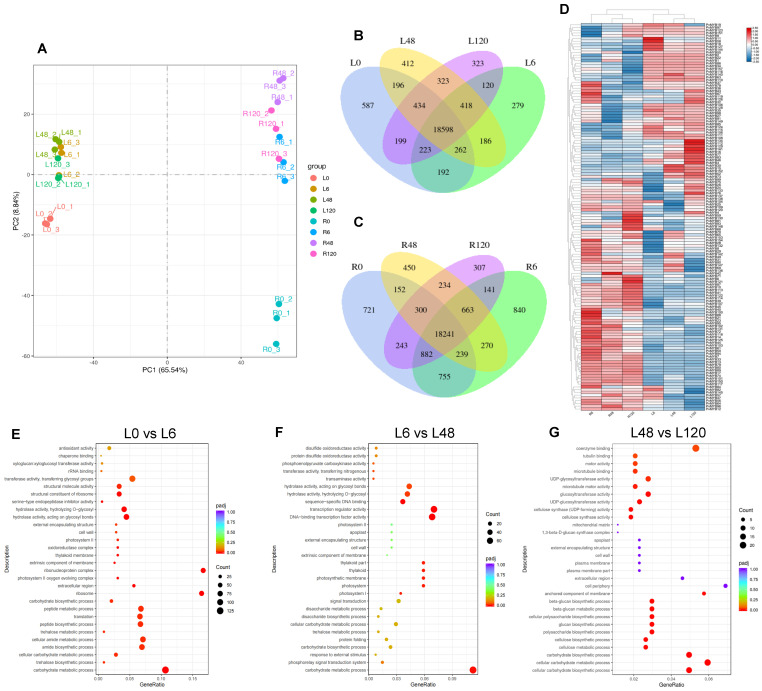
Transcriptome analysis of seashore paspalum in response to salt stress. (**A**) Principal Component Analysis (PCA) of transcriptome data, explaining 74.38% of total variance, with clear separation among treatment groups. (**B**,**C**) Venn diagrams depicting differential gene expression (DEGs) overlap in leaf and root samples at 0, 6, 48, and 120 h post-treatment. (**D**) Heatmap of *PvMYB* gene expression in leaves and roots under 0.2 mol/L NaCl, with red and blue indicating upregulation and downregulation, respectively. (**E**–**G**) Temporal dynamics of GO functional enrichment in seashore paspalum leaf transcriptomes under salt stress. Data provide insights into the temporal adaptive mechanisms of seashore paspalum under salt stress.

Principal component analysis (PCA) and differentially expressed genes (DEG) analysis were employed to elucidate the transcriptome dynamics of seashore paspalum under salinity, revealing that the expression patterns of the *PvMYB* genes and their regulatory patterns under salt stress, as shown by PCA which displayed dissimilar clustering of leaf and root data, illustrating variations in gene expression [43]. This clustering of leaf and root samples illustrated the tissue specificity of the gene expression responses to salt stress (Figure 5A). Normal analysis of the DEGs by a Venn diagram revealed further the different modes of salt stress responses regarding common and different genes at the various time points and tissue types (Figure 5B,C). In leaves, the untreated group (L0) had 587 DEGs, while for the treated groups (6 h (L6), 48 (L48), or 120 h (L120) treatment, the number of unique DEGs were 279,412 and 323, respectively. These revealed the major reprogramming of gene expression which occurs as parts of the response to salt stress, with some of the genes involved being those of an innate response of growth to salt. In roots a total of 721 unique DEGs were found in the untreated group of roots (R0), while 840, 450 and 307 unique DEGs were found in the R6, R48, and R120 treatment groups, respectively. The numbers of DEGs found in the treatments show a significant overlap which gives strength to the adaptability of the plant for its reactions to the various durations of salt stress, involving common use of some regulatory genes at all time points.

A clustered heatmap-based view of the cohort of MYB transcription factor members gave insight into expression changes under salt stress (Figure 5D). Of the total of 140 PvMYB family members which exhibited altered transcript concentration, three different time periods were tested (6 h, 48 h, and 120 h) after the application of 0.2 mol/L NaCl to indicate the strong effect osmotically induced stress has on expression of these transcription factors. This graphical representation unveiled organ-preferential profiles, wherein PvMYB47, PvMYB61, PvMYB123, and PvMYB150 manifested upregulated levels predominantly in foliar tissues, underscoring their prominence in aboveground acclimation to ionic disequilibrium. Instead, we saw that PvMYB85, PvMYB90, PvMYB123, and PvMYB150 were seen to show increased transcripts predominately in root locations indicating a role in the subterranean counter measures to NaCl treatment. It is interesting therefore that PvMYB85, PvMYB123 and PvMYB150 were all very much increased in expression in both leaves and roots, suggesting their involvement in these processes indicating that these loci may be important regulators of halotolerance in seashore paspalum.

To preliminarily investigate the transcriptional dynamics in seashore paspalum leaves in response to salt stress, we performed gene ontology (GO) enrichment analysis on differentially expressed genes (Figure 5E–G). During the early stress phase (0 h vs. 6 h), plants primarily activated antioxidant defense systems, maintaining cellular homeostasis through upregulation of molecular chaperone function and remodeling of primary metabolism. Progressing into the mid-stress phase (6 h vs. 48 h), response mechanisms shifted toward enhanced hydrolase activity and reinforced transcriptional regulatory networks to accelerate clearance of damaged proteins and signal transduction. During the late stress phase (48 h vs. 120 h), the system concentrated on optimizing coenzyme binding efficiency and dynamic reorganization of the microtubule cytoskeleton, thereby safeguarding restoration of cell division and elongation functions. This dynamic regulatory strategy exemplifies the molecular basis through which seashore paspalum adapts to chronic salt stress via staged, specific physiological and biochemical remodeling. However, GO enrichment analysis provides only preliminary functional classification hypotheses and is insufficient to delineate the precise molecular mechanisms of salt tolerance. Future studies incorporating functional validation of key genes, quantification of metabolic pathway intermediates, and analysis of protein interaction networks will be required to elucidate the temporal regulatory network underlying salt tolerance in seashore paspalum.

### 2.6. Prediction of PvMYB Protein–Protein Interaction Network in Seashore Paspalum

**Figure 6:**
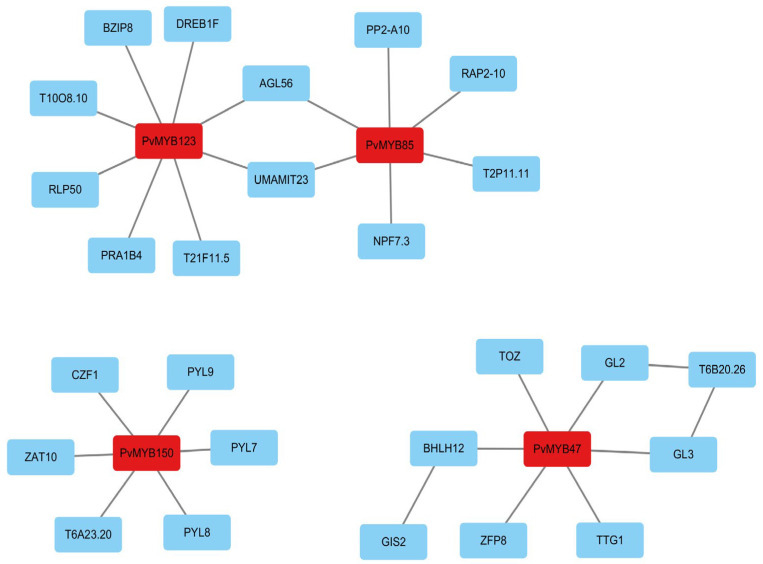
PPI Network of PvMYB Proteins. The protein–protein interaction network illustrates the associations between different PvMYB protein and other protein. PvMYB proteins are shown in red, while other protein is shown in blue.

To obtain preliminary predictive information regarding potential interactions among PvMYB transcription factors, we performed homology-based comparative analysis of PvMYB47, PvMYB85, PvMYB123, and PvMYB150 using Arabidopsis protein interaction data from the STRING database [44] to construct a hypothetical interaction network (Supplementary Table S4). The help of the PPI analysis through the STRING database has gained a predicted connection map of these abiotic stress related *MYB* genes (Figure 6) where complex associations are evident between PvMYBs and related polypeptides. In particular, the PvMYB123 has associations with various regulatory factors such as BZIP8 [4], DREB1F [45] and PP2-A10, which are known to control important features of osmotic adaptation. In consequence, associations between PvMYB85 and associates such as RAP2-10 and NPF7.3 are presumably involved in the maintenance of homeostatic balances of water status and ionic flows within the halophyte plant. Furthermore, the interaction of PvMYB150 with genes such as CZF1 and PYL9 [46] suggests its possible interaction with the plant hormone signaling systems, especially related to the regulation of the ABA pathway. However, these in silico predictions require experimental validation via yeast two-hybrid and co-immunoprecipitation (Co-IP) assays. These results are retained as testable hypotheses for future functional investigations.

### 2.7. Identification and Analysis of Key MYB Regulatory Genes

**Figure 7:**
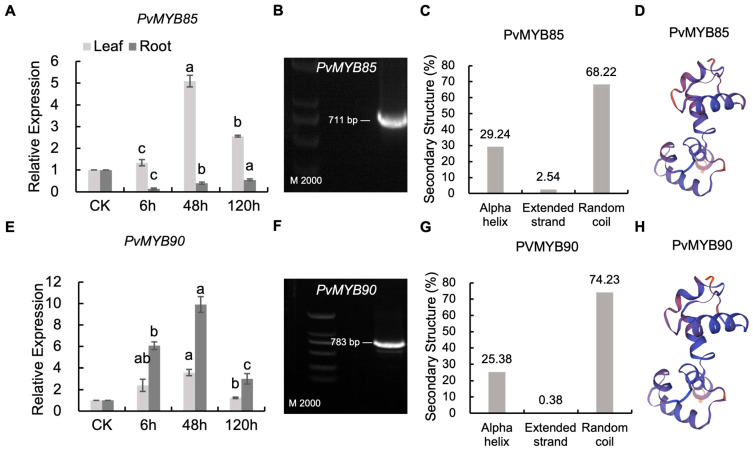
Comprehensive molecular characterization of PvMYB85 and PvMYB90. (**A**,**E**) Relative expression levels determined by qRT-PCR in leaves and roots under salt stress (0, 6, 48, and 120 h post-treatment), a, b, c represents the significance. (**B**,**F**) Agarose gel electrophoresis of PCR products from cloned CDS; expected amplicon sizes: 711 bp for PvMYB85 and 783 bp for PvMYB90. M indicates DNA marker (2000 bp). (**C**,**G**) Predicted secondary structure composition analyzed. (**D**,**H**) Three-dimensional protein models generated by SWISS-MODEL.

To quantitatively cross-validate our transcriptomic dataset, we performed targeted qRT-PCR analysis of PvMYB85 and PvMYB90 using stringent criteria. The results indicated that expression levels of *PvMYB85* and *PvMYB90* in leaves were upregulated under salt stress, the highest expression of both occurring at the 48 h. In roots the expression of *PvMYB90* was also upregulated under salt stress, peaking at 48 h. This independent validation experiment further substantiated the reliability of our transcriptomic data, confirming that PvMYB85 and PvMYB90 exhibit time-dependent regulatory roles during salt stress responses (Figure 7A,D). The *PvMYB85* and *PvMYB90* genes sequences were obtained from NCBI [47]. Their secondary and tertiary protein structures were predicted based on the amino acid sequences of these proteins. The secondary structure analysis revealed that the PvMYB85 protein consists of 29.24% α-helices, 2.54% extended strands, and 68.22% random coils. In contrast, the PvMYB90 protein contains 25.38% α-helices, 0.38% extended strands, and 74.23% random coils (Figure 7C,G). The three-dimensional protein models of PvMYB85 and PvMYB90 were constructed using the SWISS-MODEL (https://swissmodel.expasy.org, accessed on 17 October 2025) online software [48] (Figure 7D,H). To ensure amplification specificity, primers were designed to target unique regions within the PvMYB85 and PvMYB90 coding sequences, predicted to yield amplicons of 711 bp and 783 bp, respectively. Successful amplification was demonstrated by agarose gel electrophoresis of two independent biological replicates, which consistently showed prominent bands at the expected sizes (Figure 7B,F). Minor lower-intensity bands observed in some lanes represent primer dimers—a common occurrence at high PCR cycle numbers—that did not compromise cloning efficiency. Crucially, the identity of each primary amplicon was authenticated by Sanger sequencing, confirming unambiguous amplification of the target genes. Collectively, these findings support PvMYB85 and PvMYB90 as core candidate genes regulating time-dependent salt stress responses. Subsequent functional validation through the construction of overexpression lines and gene-edited mutants will be conducted to elucidate their molecular regulatory mechanisms.

### 2.8. Subcellular Localization of PvMYB85 and PvMYB90 Proteins

**Figure 8:**
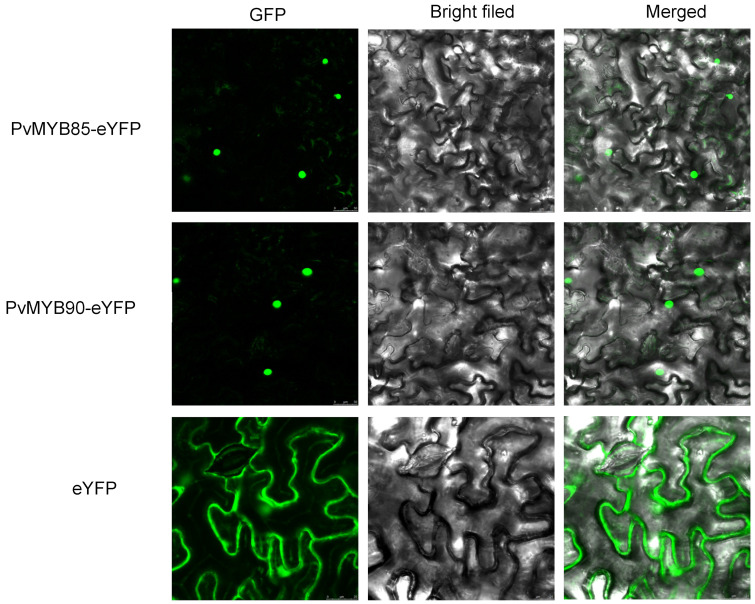
Subcellular localization of PvMYB85 and PvMYB90 proteins. GFP (first image) shows the green fluorescence image. Bright field (second image) represents the bright field image, and the merged (third image) displays the combined green fluorescence and bright field images.

Subcellular localization studies of PvMYB85 and PvMYB90 proteins can provide significant clues to the protein’s function. Observation of subcellular localization was conducted using a laser confocal microscope. The positive control consisted of tobacco leaves transformed with the pFGC-eYFP empty vector, while the experimental group comprised tobacco leaves transformed with the pFGC -PvMYB85-eYFP and pFGC -PvMYB90-eYFP expression vector. The results demonstrated that in tobacco leaves, the fluorescence signal from the empty vector was detected in both the cytoplasm and the nucleus. In contrast, the PvMYB85 and PvMYB90 proteins exhibited a strong fluorescence signal localized exclusively in the nucleus (Figure 8). Thus, the subcellular localization of PvMYB85 and PvMYB90 proteins in the nucleus is consistent with prior predictions. This confirmation of nuclear targeting reinforces their anticipated functions as transcription factors, likely modulating downstream gene expression to enhance salt tolerance mechanisms in seashore paspalum.

## 3. Discussion

### 3.1. Evolutionary Dynamics and Functional Diversification of the MYB Family

The MYB transcription factor superfamily is one of the largest and most functionally versatile gene families in plants, playing crucial roles in various biological processes, including cell differentiation, secondary metabolism, and responses to abiotic stresses [27]. In addition to salt stress, its regulatory function in abiotic stresses such as drought [49], extreme temperature [50], and oxidative stress [51] has been widely confirmed. In seashore paspalum, a halophytic turfgrass known for its remarkable salt tolerance, we identified 157 *PvMYB* genes, a number comparable to rice (*Oryza sativa*, 155) and oil palm (*Elaeis guineensis*, 159), but significantly different from other species, such as mango (*Mangifera indica*, 54) and pearl millet (*Pennisetum glaucum*, 208) [52,53,54,55,56]. This variation in gene family size reflects evolutionary expansion and functional diversification driven by lineage-specific adaptations, possibly as a response to saline-alkaline environments.

Phylogenetic analysis revealed that the PvMYB family is divided into several clades, with each clade exhibiting similar intron-exon organization and conserved motif structures. Such intra-clade conservation and inter-clade divergence likely reflect subfunctionalization and neofunctionalization during evolutionary adaptation [57,58]. Similar expansion patterns have been observed in other halophytic grasses, such as *Salicornia brachiata* Roxb. and *Sorghum bicolor* [59,60], suggesting that MYB diversification plays a pivotal role in shaping the stress tolerance mechanisms of halophytes. Similarly, other gene families also play crucial roles in regulating plant salt tolerance. For instance, overexpression of the NAC family member *FvNAC29* from wild strawberry (*Fragaria vesca*) significantly enhances salt and cold tolerance in *Arabidopsis* by regulating stress-responsive genes such as *AtRD29a* and *AtP5CS1* [61]; heterologous expression of the WRKY transcription factor *VhWRKY44* from grape also effectively improves dual resistance to both cold and salt in *Arabidopsis* [62]. Additionally, the AP2/ERF gene family contributes to salt tolerance by participating in stress response mechanisms in species such as *Tritipyrum* [63]. Beyond salinity, transcription factors contribute to tolerance against other abiotic stresses, such as drought and cold. Overexpression of *MbICE3* in lettuce significantly enhanced cold and drought tolerance by upregulating antioxidant enzymes (SOD and POD) and positively regulating the *LsCBF* gene [64]. Similarly, heterologous expression of *MbWRKY50* in tomato conferred enhanced cold and drought resistance by activating downstream target genes such as *LeABI3*, *LeNCED1*, and *LeCBF1/3*, synergistically enhancing SOD and POD activities to strengthen ROS scavenging [65]. Collectively, these studies collectively reveal the core role of transcription factors in coordinating the regulation of antioxidant defense and stress response, providing important molecular breeding strategies for the study of stress resistant crop varieties.

### 3.2. Regulatory Mechanisms and Cis-Regulatory Elements in PvMYB Genes

To investigate the regulatory mechanisms, we analyzed the 2000 bp upstream promoter regions of *PvMYB* genes using the PlantCARE database. This approach, commonly used in genome-wide studies of non-model organisms, identified light-responsive cis-regulatory elements as predominant in the promoters of *PvMYB* genes. Promoter analysis revealed that *PvMYB* genes contain numerous cis-elements responsive to light, abscisic acid (ABA), gibberellins (GA), methyl jasmonate (MeJA), and auxins. These motifs indicate that *PvMYB* genes are tightly regulated by both environmental and hormonal signals. The enrichment of light- and stress-responsive elements suggests an interplay between photoperiodic regulation and stress signaling, which may fine-tune the plant’s photosynthetic adaptation to saline conditions [66].

While promoter analysis is a widely accepted method [67,68,69], we acknowledge that biases may arise due to the lack of precise transcription start site (TSS) mapping. Future studies utilizing techniques like 5′RACE or CAGE-seq will provide more accurate mapping of cis-elements and TSSs, particularly for key genes such as *PvMYB85* and *PvMYB90*. qRT-PCR validation demonstrated peak expression of PvMYB85 and PvMYB90 at 48 h post salt treatment, supporting transcriptomic data. Subcellular localization assays confirmed the nuclear localization of these proteins, reinforcing their predicted roles as transcriptional regulators.

### 3.3. Integrated Molecular Responses to Salt Stress: Expression, GO, and PPI Insights

Integrated transcriptomic, gene ontology (GO), and protein–protein interaction (PPI) analyses underscored the pivotal role of *PvMYB* genes in regulating salt stress responses. Several PvMYB members, including PvMYB85, PvMYB123, and PvMYB150, were significantly upregulated in roots and leaves under saline conditions, confirming their involvement in salt tolerance mechanisms. Early stage responses (6 h) primarily involved the regulation of antioxidant genes and cell wall biosynthesis, mitigating oxidative damage—consistent with MYB-mediated activation of reactive oxygen species (ROS)-scavenging pathways in angiosperms. GO enrichment analysis revealed that these MYB proteins participate in pathways associated with “transcriptional regulation,” “response to hormone stimulus,” and “cell wall organization,” findings that align with previous studies in rice and *Arabidopsis thaliana* [70,71].

Predicted protein–protein interactions (PPIs) revealed potential associations between PvMYB proteins and key regulators, including bZIP8 (involved in ABA-dependent signaling) [7], DREB1F (a dehydration-responsive element-binding factor activating stress-responsive effectors) [24], RAP2-10 (an ERF family member enhancing osmotic adjustment), and PYL9 (an ABA receptor modulating ion homeostasis) [46]. Notably, the interactions between PvMYB123 and DREB1F, and PvMYB150 and PYL9, suggest coordinated regulation through both ABA-dependent and ABA-independent pathways, contributing to desiccation tolerance and ion balance maintenance. Furthermore, PPI network predictions indicated potential interactions between PvMYB123 and additional stress-responsive regulators, such as DREB1F, bZIP8, and RAP2-10, all of which are involved in ABA-mediated and dehydration-induced signaling pathways [72,73]. These findings imply that PvMYB factors may act as transcriptional integrators linking ABA signaling with ROS detoxification [74], thereby enhancing stress resilience in seashore paspalum.

Nevertheless, these predicted interactions require experimental validation. Future work employing yeast two-hybrid, bimolecular fluorescence complementation (BiFC), and co-immunoprecipitation assays will be necessary to confirm direct MYB–DREB and MYB–ABF interactions and elucidate their roles in salt stress signaling networks.

## 4. Materials and Methods

### 4.1. Identification of the MYB Gene Family Members in Seashore Paspalum

Genome assemblies and annotations for seashore paspalum were obtained from Phytozome (v13, JGI) (Phytozome genome ID: 672, https://phytozome-next.jgi.doe.gov/info/Pvaginatum_v3_1, accessed on 17 October 2025); NCBI taxonomy ID: 158149) [75]. Reference MYB protein sequences from *Arabidopsis thaliana* were retrieved from PlantTFDB (v5.0) [76]. Putative MYB family members in seashore paspalum were identified by performing BLASTP searches [77] (E-value threshold: 1 × 10^−5^) against the Arabidopsis reference set using TBtools (v1.108) software [42]. Candidate sequences were further validated by reciprocal BLASTP alignment against the SwissProt database (UniProt release 2023_02) [78]. Conserved MYB domains were authenticated using the NCBI Conserved Domain Search tool [47]; sequences lacking complete MYB domains (coverage < 70%) or containing premature stop codons were excluded from further analysis. This stringent curation pipeline resulted in a final catalog of 157 high-confidence PvMYB loci (Supplementary Table S1).

### 4.2. Construction of the Phylogenetic Tree for the MYB Gene Family in Seashore Paspalum

The complete repertoire of predicted MYB protein sequences from seashore pas-palum was retrieved for analysis. Multiple sequence alignment of the full-length amino acid sequences of 157 PvMYB and 117 rice MYB transcription factors was performed using the ClustalW algorithm implemented in MEGA11 (v0.13) software [39], from this alignment, a phylogenetic tree was generated via the Maximum Likelihood framework. Finally, the resulting tree underwent optimization and graphical rendering on the iTOL v4 online platform (https://itol.embl.de/, accessed on 18 December 2025) [79].

### 4.3. Physicochemical Property Analysis of the MYB Gene Family in Seashore Paspalum

Physicochemical characteristics of the proteins were computed with the ExPASy Proteomics platform (Swiss Bioinformatics Resource Portal) [80] using ProtParam and ProtScale modules. Evaluations covered aspects such as amino acid makeup (aa), estimated molecular mass (MW), predicted isoelectric point (pI), atomic formula, and the overall hydrophilic versus hydrophobic profiles of these polypeptides. The subcellular localization of PvMYB proteins was predicted using WoLF PSORT (a protein localization predictor) [40]. Protein sequences were submitted to the WoLF PSORT web server (https://wolfpsort.hgc.jp/, accessed on 10 October 2025), and predictions were based on the “plant” mode with default parameters. Proteins scoring ≥ 8.0 for nuclear localization were classified as nuclear-targeted proteins.

### 4.4. Motif Analysis and Domain Visualization of MYB Proteins in Seashore Paspalum

To identify conserved motifs within MYB transcription factors, we scanned protein sequences using the MEME Suite (v5.5.0) [41], specifying detection of 10 de novo motifs (E-value < 0.001, motif width 6–50 aa). Distribution patterns were visualized using TBtools (v1.108) [42] with default parameters for motif mapping and annotation.

### 4.5. Analysis of Cis-Acting Elements in the Promoters of the MYB Gene Family in Seashore Paspalum

Promoter sequences comprising 2000 base pairs upstream from the ATG start codon of *MYB* transcription factor genes in seashore paspalum were retrieved from the genome assembly via TBtools (v1.108) and classified as the upstream regulatory regions for these genes [42]. Subsequently, the retrieved sequences were analyzed for cis-regulatory elements through submission to the PlantCARE server [81]. Among the detected elements, those linked to phytohormone responsiveness, environmental stresses, and cellular growth or development were prioritized, with their positional mapping along the promoters illustrated using TBtools (v1.108) [42] under standard parameters.

### 4.6. Intraspecific Collinearity Analysis and Chromosomal Localization of PvMYB Genes in Seashore Paspalum

Utilizing the annotation data for the 157 *MYB* genes pinpointed in seashore paspalum, and conducted collinearity assessment and chromosome mapping through the One Step McScanX page in TBtools (v1.108) software [42].

### 4.7. Construction of the Protein–Protein Interaction (PPI) Network for PvMYB Proteins in Seashore Paspalum

Using the established MYB protein interactions in Arabidopsis thaliana, the MYB protein–protein interaction (PPI) networks were assembled for PvMYB proteins on the STRING v11 platform [44]. Networks created were then mapped in Cytoscape (v. 3.10.2) [82] with node size and colors adjusted in proportion to degree centrality to demonstrate connectivity patterns.

### 4.8. Salt Stress Treatment of Seashore Paspalum

Plant material consisted of seashore paspalum (*Paspalum vaginatum* ‘SeaSpray’) clonally propagated from stolons of a single mother plant. After 12 weeks of hydroponic culture in Hoagland nutrient solution, uniformly developed clones were selected for subsequent experiments, ensuring high genetic consistency. Modified Hoagland’s solution was used as the nutrient solution for hydroponic culture. Stock solutions were prepared using analytical-grade reagents dissolved in deionized water. Solution A contained 4 mM Ca(NO_3_)_2_·4H_2_O and 4 mM KNO_3_; Solution B contained 2 mM MgSO_4_·7H_2_O and 1 mM NH_4_H_2_PO_4_; Solution C (micronutrients) contained 2.86 g·L^−1^ H_3_BO_3_, 1.614 g·L^−1^ MnSO_4_·H_2_O, 0.22 g·L^−1^ ZnSO_4_·7H_2_O, 0.08 g·L^−1^ CuSO_4_·5H_2_O, and 0.02 g·L^−1^ (NH_4_)_4_Mo_7_O_24_·4H_2_O. Solution D was prepared by dissolving 7.485 g EDTA-Na_2_·2H_2_O and 5.561 g FeSO_4_·7H_2_O in 1 L of water and heating to 70 °C until complete chelation. Prepared stock solutions A, B, C, and D were diluted 200-fold, 200-fold, 1000-fold, and 200-fold, respectively, for use. The pH was adjusted to 6.0 ± 0.2 with 1 M KOH and verified using a calibrated pH meter (Thermo Fisher Scientific, Waltham, MA, USA). The nutrient solution was replaced every 7 days to prevent nutrient depletion. All stock solutions were stored at 4 °C and freshly prepared monthly. The experiment utilized 28 cm × 22 cm hydroponic pots with a planting density of 20 plants per pot (each plant established from 10 stolon cuttings), 3 L of Hoagland nutrient solution per pot, and six pots total. The photoperiod consisted of 16 h of light and 8 h of darkness, the temperatures during the day were maintained at 30 °C and 25 °C at night during the whole growing performance. The daytime humidity was maintained at 50% and the nighttime humidity at 70%. These conditions were instrumental in achieving satisfactory plant growth.

After the twelve-week period of growing the seashore paspalum, various treatments of a stress character were applied. The treatment batches received a treatment of 0.2 M NaCl added to the Hoagland mixture which caused a salinity treatment. The six hydroponic pots were randomly divided into three biological replicates, with two pots per replicate. Samples (0.2 g each of leaf and root tissues) were rapidly collected at four time points: before salt stress treatment (0 h) and at 6 h, 48 h, and 120 h post-treatment. Each tissue sample was prepared with three technical replicates. All samples were immediately snap-frozen in liquid nitrogen and stored at −80 °C. During this salt treatment, there were some visible changes in the leaves. At 6 h after the treatment the leaves wilted some but were extended. After a treatment of 48 h, there was visible indication of withering and curling of the leaves. At the end of 120 h there was excessive curling of the leaves, while the older leaves began turning yellow and breaking down.

### 4.9. Comprehensive Transcriptome and MYB Transcription Factor Analysis in Seashore Paspalum Under Salt Stress

The objective of this investigation was to clarify the transcriptomic profiles of seashore paspalum subjected to salinity challenge. High-throughput RNA sequencing outputs from foliar and radicular samples, treated with NaCl over durations of 0, 6, 48, and 120 h, underwent comprehensive evaluation. Expression profiles were visualized as heatmaps using TBtools (v1.108) [42,83,84], and differentially expressed transcripts (DEGs) were identified from triplicate biological samples using DESeq2 [85] with thresholds of |log_2_FC| > 2 and FDR < 0.01 [86]. In addition, Gene Ontology (GO) annotation enrichment was conducted following submission of full-length PvMYB polypeptides to the eggNOG-mapper platform [87], with enriched categories subsequently illustrated in a GO scatter plot [88]. Taken together, these multifaceted approaches yield valuable insights into the regulatory interactions and biological functions of MYB transcription factors during salinity stress in seashore paspalum.

### 4.10. Real-Time Quantitative PCR and Gene Cloning

Frozen leaf and root tissues of seashore paspalum underwent dual pulverization in a Cryogenic Grinder (JXFSTPRP-CLN, Shanghai, China). Isolation of total RNA from these preparations relied on the Plant Total RNA Extraction Kit (TaKaRa, Tokyo, Japan), following the vendor’s prescribed protocol. Synthesis of complementary DNA (cDNA) employed the PrimeScript RT Reagent Kit, augmented by gDNA Eraser (TaKaRa, Tokyo, Japan). Assessments of RNA purity and structural wholeness involved the Agilent 2100 Bioanalyzer (Agilent Technologies, Santa Clara, CA, USA), NanoDrop 2000 spectrophotometer (Thermo Fisher Scientific, Waltham, MA, USA), and agarose gel electrophoresis. Genomic DNA removal was conducted by mixing 2 μL of 8× gDNA Eraser Premix with 1 μg of total RNA and RNase-free water (total volume 16 μL), then incubating at 42 °C for 2 min. Reverse transcription was subsequently initiated by adding 4 μL of 5× RT Premix to the treated RNA mixture (final volume 20 μL), followed by incubation at 37 °C for 10 min for cDNA synthesis, heat inactivation at 85 °C for 5 s, and cooling to 4 °C. The thermal cycling protocol was performed on a CFX96 Real-Time PCR Detection System (Bio-Rad, Hercules, CA, USA) using TB Green Premix Ex Taq II (TaKaRa, Tokyo, Japan). Reaction mixtures (20 μL) contained 2 μL of diluted cDNA, 10 μL of 2× SYBR Premix, 0.4 μL of each primer (10 μM), and 7.2 μL of RNase-free water. Specific qRT-PCR primers were designed using Primer3Plus with the seashore paspalum actin gene (PvActin) as the internal reference (Table 1) [89]. Fluorescence signals were acquired at the 60 °C extension step during each cycle. A melting curve analysis was automatically generated post-PCR (65–95 °C, increment 0.5 °C/5 s) to verify primer specificity. Each sample was analyzed in triplicate technical replicates, with Ct values calculated using the instrument’s default threshold setting. No-template controls (NTC) and no-reverse-transcription controls (NRT) were included in each run to ensure absence of contamination and genomic DNA carryover, respectively. Variations in transcript levels were quantified via the 2^−ΔΔCt^ approach [90].

Total RNA underwent reverse transcription to generate first-strand cDNA employing the PrimeScript™ II 1st Strand cDNA Synthesis Kit (TaKaRa, Tokyo, Japan), in accordance with the supplier’s guidelines. Primer pairs, encompassing forward and reverse variants, were formulated to amplify the PvMYB85 coding region (CDS), drawing from the archived MYB85 and MYB90 mRNA sequence of seashore paspalum in the NCBI repository (Table 1). Amplification via PCR proceeded in 50-μL volumes comprising 25 μL 2× Phanta Flash Master Mix (Dye Plus), 2 μL template cDNA, 2 μL each of forward (F) and reverse (R) primers, plus 19 μL nuclease-free H_2_O. Annealing occurred at 60 °C, with cycling parameters adhering to the routine outlined in the Vazyme user guide.

### 4.11. Subcellular Localization Assay

The coding sequence (CDS) of *PvMYB85* and *PvMYB90* was cloned into the pFGC-eYFP expression cassette, harboring the enhanced yellow fluorescent protein (eYFP) reporter. Amplification primers included pFGC-eYFP-F and pFGC-eYFP-R (Table 1), with conditions identical to those reported in Section 4.10. Vector construction was verified by colony PCR screening, restriction digestion with BamHI, and Sanger sequencing to confirm in-frame fusion and sequence fidelity before downstream applications. The pFGC-PvMYB85-eYFP and pFGC-PvMYB90-eYFP constructs and the empty pFGC-eYFP backbone was then electroporated into Agrobacterium tumefaciens GV3101 electrocompetent strains. Agrobacterium cells were harvested by centrifugation and resuspended in infiltration buffer supplemented with 100 μmol/L acetosyringone to a final OD_600_ of 0.6–0.8 for transient transformation of tobacco leaves. Following resuspension, the bacterial suspensions were infiltrated into the lower epidermis of *Nicotiana benthamiana* leaves for agroinfiltration-based transient assay [91]. The leaves were treated for 48 h post infiltration, in a growth chamber, prior to investigation by laser scanning confocal microscopy for investigation of pFGC-PvMYB85-eYFP and pFGC-PvMYB90-eYFP chimeric protein expression and targeting. Confocal microscopy settings were standardized using an Agilent TCS SP5 II laser scanning confocal microscope equipped with a 40× water immersion objective (Agilent, USA). Green fluorescence was excited at 561 nm using a DPSS laser (30% power) and detected at 525–560 nm. All images were acquired with pinhole set to 1 Airy unit, pixel resolution of 1024 × 1024, and pixel dwell time of 1.58 μs. Consistent acquisition parameters were applied across all samples to ensure quantitative comparability.

### 4.12. Data Analysis

To promote reliable and consistent results in this study, three different isolations of RNA were utilized each with replicate biological samples of three. One-way analysis of variance (ANOVA) was carried out on these repeated results. All statistical analyses were conducted using IBM SPSS Statistics for Windows, Version 26.0. Differences were regarded as significant when *p* < 0.05 or highly significant when *p* < 0.01. Results of assays will be seen as averages ± stand deviation (SD). Duncan’s multiple range tests (*p* < 0.05) were used to distinguish from the treatment means.

## 5. Conclusions

This study provides the first comprehensive characterization of the MYB transcription factor family in seashore paspalum. Through the integration of evolutionary, transcriptomic, and protein interaction data, we identified both conserved and specialized *MYB* genes that mediate salt stress responses. The identification and validation of PvMYB85 and PvMYB90 offer valuable genetic resources for dissecting the molecular mechanisms underlying salt tolerance in halophytic grasses.

Future research using overexpression, gene knockout, and ChIP-seq approaches will be essential to uncover the downstream targets of these transcription factors and construct a comprehensive regulatory network of MYB-mediated salt tolerance. Such insights will not only enhance our understanding of halophyte adaptation but also provide a molecular basis for breeding salt-tolerant turfgrass and cereal crops to improve agricultural productivity in saline–alkaline environments.

## Figures and Tables

**Table 1:** Primer sequences for target-gene amplification and vector confirmation.

Primer	Primer Sequence (5′–3′)
Pv-Actin-F	CTTCTCTCAGCACTTTCCAACA
Pv-Actin-R	AAACATAACCTGCAATCTCTCC
PvMYB85-F	ATGGTGACTGTGAGAGAGGAGGT
PvMYB85-R	TTATTTACCATAACCAAATTGTGGAGCCAAC
PvMYB90-F	ATGGGGAGGGCTCCGTG
PvMYB90-R	CTAAATCTGCGGCAATTCTTGGTGC
pFGC-eYFP-F	ATCCTTCGCAAGACCCTTCCT
pFGC-eYFP-R	GGACACGCTGAACTTGTGGC

## Data Availability

The datasets generated and analyzed during the current study are not publicly available given the restrictions to data sharing imposed, but de-identified data are available from the corresponding author on reasonable request.

## Supplementary Materials

The following supporting information can be downloaded at: https://www.mdpi.com/article/10.3390/ijms27021068/s1.

## References

[1] Zhang H., Lang Z., Zhu J.-K., Wang P. (2025). Tackling Abiotic Stress in Plants: Recent Insights and Trends. Stress Biol..

[2] Varadharajan V., Rajendran R., Muthuramalingam P., Runthala A., Madhesh V., Swaminathan G., Murugan P., Srinivasan H., Park Y., Shin H. (2025). Multi-Omics Approaches Against Abiotic and Biotic Stress—A Review. Plants.

[3] Acosta-Motos J., Ortuño M., Bernal-Vicente A., Diaz-Vivancos P., Sanchez-Blanco M., Hernandez J. (2017). Plant Responses to Salt Stress: Adaptive Mechanisms. Agronomy.

[4] Fang S., Hou X., Liang X. (2021). Response Mechanisms of Plants Under Saline-Alkali Stress. Front. Plant Sci..

[5] Ahmed M., Tóth Z., Decsi K. (2024). The Impact of Salinity on Crop Yields and the Confrontational Behavior of Transcriptional Regulators, Nanoparticles, and Antioxidant Defensive Mechanisms under Stressful Conditions: A Review. Int. J. Mol. Sci..

[6] Frene J.P., Pandey B.K., Castrillo G. (2024). Under Pressure: Elucidating Soil Compaction and Its Effect on Soil Functions. Plant Soil.

[7] Liu H., Tang X., Zhang N., Li S., Si H. (2023). Role of bZIP Transcription Factors in Plant Salt Stress. Int. J. Mol. Sci..

[8] Liu J., Chen X., Chen W., Zhang Y., Wang A., Zheng Y. (2023). Ecosystem Service Value Evaluation of Saline—Alkali Land Development in the Yellow River Delta—The Example of the Huanghe Island. Water.

[9] Gupta B., Huang B. (2014). Mechanism of Salinity Tolerance in Plants: Physiological, Biochemical, and Molecular Characterization. Int. J. Genom..

[10] Deinlein U., Stephan A.B., Horie T., Luo W., Xu G., Schroeder J.I. (2014). Plant Salt-Tolerance Mechanisms. Trends Plant Sci..

[11] Shokri N., Hassani A., Sahimi M. (2024). Multi-Scale Soil Salinization Dynamics from Global to Pore Scale: A Review. Rev. Geophys..

[12] Zhang C., Liu X. (2025). Effects of Salt Stress on Growth and Physiological Characteristics of *Chamerion angustifolium* Seedlings. Front. Plant Sci..

[13] Greco E., Talarico E., Guarasci F., Camoli M., Palermo A.M., Zambelli A., Chiappetta A., Araniti F., Bruno L. (2025). Epigenetic Mechanisms of Plant Adaptation to Cadmium and Heavy Metal Stress. Epigenomes.

[14] Antoniou C., Chatzimichail G., Xenofontos R., Pavlou J.J., Panagiotou E., Christou A., Fotopoulos V. (2017). Melatonin Systemically Ameliorates Drought Stress-induced Damage in *Medicago sativa* Plants by Modulating Nitro-oxidative Homeostasis and Proline Metabolism. J. Pineal Res..

[15] Bai Y., Zhang T., Zheng X., Li B., Qi X., Xu Y., Li L., Liang C. (2023). Overexpression of a WRKY Transcription Factor McWRKY57-like from Mentha *Canadensis* L. Enhances Drought Tolerance in Transgenic Arabidopsis. BMC Plant Biol..

[16] Xu L., Yang L., Li A., Guo J., Wang H., Qi H., Li M., Yang P., Song S. (2024). An AP2/ERF Transcription Factor Confers Chilling Tolerance in Rice. Sci. Adv..

[17] Yu Y., Wu Y., He L. (2023). A Wheat WRKY Transcription Factor TaWRKY17 Enhances Tolerance to Salt Stress in Transgenic *Arabidopsis* and Wheat Plant. Plant Mol. Biol..

[18] Wu J., Yu C., Huang L., Gan Y. (2021). A Rice Transcription Factor, *OsMADS57*, Positively Regulates High Salinity Tolerance in Transgenic *Arabidopsis thaliana* and *Oryza sativa* Plants. Physiol. Plant..

[19] Rahayu R., Ariyanto D.P., Usrotin A.H., Hatami F.R., Mo Y.G. (2023). Assessment of Turf Quality in *Paspalum vaginatum* Sw. Accessions of Sumatra, Java, and Bali (Indonesia) with Clay and Amended Sand Growing Media. Biodiversitas.

[20] Wang H., Fang T., Li X., Xie Y., Wang W., Hu T., Kudrna D., Amombo E., Yin Y., Fan S. (2024). Whole-genome Sequencing of Allotetraploid Bermudagrass Reveals the Origin of *Cynodon* and Candidate Genes for Salt Tolerance. Plant J..

[21] Lonard R.I., Judd F.W., Stalter R. (2015). Biological Flora of Coastal Dunes and Wetlands: *Paspalum vaginatum* Sw. J. Coast. Res..

[22] Wu X., Xia M., Su P., Zhang Y., Tu L., Zhao H., Gao W., Huang L., Hu Y. (2024). MYB Transcription Factors in Plants: A Comprehensive Review of Their Discovery, Structure, Classification, Functional Diversity and Regulatory Mechanism. Int. J. Biol. Macromol..

[23] Liu L., White M.J., MacRae T.H. (1999). Transcription Factors and Their Genes in Higher Plants: Functional Domains, Evolution and Regulation. Eur. J. Biochem..

[24] Erpen L., Sunitibala Devi H., Grosser J.W., Dutt M. (2017). Potential Use of the DREB/ERF, MYB, NAC and WRKY Transcription Factors to Improve Abiotic and Biotic Stress in Transgenic Plants. PCTOC.

[25] Zhang Y., Xu J., Li R., Ge Y., Li Y., Li R. (2023). Plants’ Response to Abiotic Stress: Mechanisms and Strategies. Int. J. Mol. Sci..

[26] Cao Y., Li K., Li Y., Zhao X., Wang L. (2020). MYB Transcription Factors as Regulators of Secondary Metabolism in Plants. Biology.

[27] Dubos C., Stracke R., Grotewold E., Weisshaar B., Martin C., Lepiniec L. (2010). MYB Transcription Factors in *Arabidopsis*. Trends Plant Sci..

[28] Stracke R., Werber M., Weisshaar B. (2001). The R2R3-MYB Gene Family in *Arabidopsis thaliana*. Curr. Opin. Plant Biol..

[29] Li C., Lu S. (2014). Genome-Wide Characterization and Comparative Analysis of R2R3-MYB Transcription Factors Shows the Complexity of MYB-Associated Regulatory Networks in *Salvia miltiorrhiza*. BMC Genom..

[30] Millard P.S., Kragelund B.B., Burow M. (2019). R2R3 MYB Transcription Factors—Functions Outside the DNA-Binding Domain. Trends Plant Sci..

[31] Yang J., Zhang B., Gu G., Yuan J., Shen S., Jin L., Lin Z., Lin J., Xie X. (2022). Genome-Wide Identification and Expression Analysis of the R2R3-MYB Gene Family in Tobacco (*Nicotiana Tabacum* L.). BMC Genom..

[32] Zhang X., Chen L., Shi Q., Ren Z. (2020). SlMYB102, an R2R3-Type MYB Gene, Confers Salt Tolerance in Transgenic Tomato. Plant Sci..

[33] Li Z., Peng R., Tian Y., Han H., Xu J., Yao Q. (2016). Genome-Wide Identification and Analysis of the MYB Transcription Factor Superfamily in *Solanum lycopersicum*. Plant Cell Physiol..

[34] Ren C., Li Z., Song P., Wang Y., Liu W., Zhang L., Li X., Li W., Han D. (2023). Overexpression of a Grape MYB Transcription Factor Gene VhMYB2 Increases Salinity and Drought Tolerance in *Arabidopsis thaliana*. Int. J. Mol. Sci..

[35] Li W., Wei Y., Zhang L., Wang Y., Song P., Li X., Han D. (2023). FvMYB44, a Strawberry R2R3-MYB Transcription Factor, Improved Salt and Cold Stress Tolerance in Transgenic *Arabidopsis*. Agronomy.

[36] Zhao Y., Yang Z., Ding Y., Liu L., Han X., Zhan J., Wei X., Diao Y., Qin W., Wang P. (2019). Over-Expression of an R2R3 MYB Gene, GhMYB73, Increases Tolerance to Salt Stress in Transgenic *Arabidopsis*. Plant Sci..

[37] He Y., Dong Y., Yang X., Guo D., Qian X., Yan F., Wang Y., Li J., Wang Q. (2020). Functional Activation of a Novel R2R3-MYB Protein Gene, *GmMYB68*, Confers Salt-Alkali Resistance in Soybean (*Glycine max* L.). Genome.

[38] Eddy S.R. (2011). Accelerated Profile HMM Searches. PLoS Comput. Biol..

[39] Tamura K., Stecher G., Kumar S. (2021). MEGA11: Molecular Evolutionary Genetics Analysis Version 11. Mol. Biol. Evol..

[40] Horton P., Park K.-J., Obayashi T., Fujita N., Harada H., Adams-Collier C.J., Nakai K. (2007). WoLF PSORT: Protein Localization Predictor. Nucleic Acids Res..

[41] Bailey T.L., Johnson J., Grant C.E., Noble W.S. (2015). The MEME Suite. Nucleic Acids Res..

[42] Chen C., Chen H., Zhang Y., Thomas H.R., Frank M.H., He Y., Xia R. (2020). TBtools: An Integrative Toolkit Developed for Interactive Analyses of Big Biological Data. Mol. Plant.

[43] Ma J., Zhao D., Tang X., Yuan M., Zhang D., Xu M., Duan Y., Ren H., Zeng Q., Wu J. (2022). Genome-Wide Association Study on Root System Architecture and Identification of Candidate Genes in Wheat (*Triticum aestivum* L.). Int. J. Mol. Sci..

[44] Szklarczyk D., Gable A.L., Lyon D., Junge A., Wyder S., Huerta-Cepas J., Simonovic M., Doncheva N.T., Morris J.H., Bork P. (2019). STRING V11: Protein–Protein Association Networks with Increased Coverage, Supporting Functional Discovery in Genome-Wide Experimental Datasets. Nucleic Acids Res..

[45] Ma J.-T., Yin C.-C., Guo Q.-Q., Zhou M.-L., Wang Z.-L., Wu Y.-M. (2015). A Novel DREB Transcription Factor from *Halimodendron halodendron* Leads to Enhance Drought and Salt Tolerance in *Arabidopsis*. Biol. Plant..

[46] Wang Z., Zhou J., Zou J., Yang J., Chen W. (2024). Characterization of *PYL* Gene Family and Identification of *HaPYL* Genes Response to Drought and Salt Stress in Sunflower. PeerJ.

[47] Marchler-Bauer A., Derbyshire M.K., Gonzales N.R., Lu S., Chitsaz F., Geer L.Y., Geer R.C., He J., Gwadz M., Hurwitz D.I. (2015). CDD: NCBI’s Conserved Domain Database. Nucleic Acids Res..

[48] Waterhouse A., Bertoni M., Bienert S., Studer G., Tauriello G., Gumienny R., Heer F.T., de Beer T.A.P., Rempfer C., Bordoli L. (2018). SWISS-MODEL: Homology Modelling of Protein Structures and Complexes. Nucleic Acids Res..

[49] Jian L., Kang K., Choi Y., Suh M.C., Paek N. (2022). Mutation of *OsMYB60* Reduces Rice Resilience to Drought Stress by Attenuating Cuticular Wax Biosynthesis. Plant J..

[50] Lv Y., Yang M., Hu D., Yang Z., Ma S., Li X., Xiong L. (2017). The OsMYB30 Transcription Factor Suppresses Cold Tolerance by Interacting with a JAZ Protein and Suppressing *β*-Amylase Expression. Plant Physiol..

[51] Upadhyaya G., Das A., Ray S. (2021). A Rice R2R3-MYB (*OsC1*) Transcriptional Regulator Improves Oxidative Stress Tolerance by Modulating Anthocyanin Biosynthesis. Physiol. Plant..

[52] Zhou L., Yarra R., Jin L., Cao H. (2020). Genome-Wide Identification and Expression Analysis of MYB Gene Family in Oil Palm (*Elaeis guineensis* Jacq.) under Abiotic Stress Conditions. Environ. Exp. Bot..

[53] Zhang H., Liu Z., Luo R., Sun Y., Yang C., Li X., Gao A., Pu J. (2022). Genome-Wide Characterization, Identification and Expression Profile of MYB Transcription Factor Gene Family during Abiotic and Biotic Stresses in Mango (*Mangifera indica*). Plants.

[54] Ji K., Liu C., Wu K., Yue Z., Dong Y., Gong B., Xu Y. (2023). Genome-Wide Characterization of the R2R3-MYB Gene Family in *Diospyros oleifera*. Agriculture.

[55] Zhang H.-C., Gong Y.-H., Tao T., Lu S., Zhou W.-Y., Xia H., Zhang X.-Y., Yang Q.-Q., Zhang M.-Q., Hong L.-M. (2024). Genome-Wide Identification of R2R3-MYB Transcription Factor Subfamily Genes Involved in Salt Stress in Rice (*Oryza sativa* L.). BMC Genom..

[56] Katiyar A., Smita S., Lenka S.K., Rajwanshi R., Chinnusamy V., Bansal K.C. (2012). Genome-Wide Classification and Expression Analysis of MYB Transcription Factor Families in Rice and *Arabidopsis*. BMC Genom..

[57] Żyła N., Babula-Skowrońska D. (2023). Evolutionary Consequences of Functional and Regulatory Divergence of HD-Zip I Transcription Factors as a Source of Diversity in Protein Interaction Networks in Plants. J. Mol. Evol..

[58] Shi X., Yang T., Ren M., Fu J., Bai J., Cui H. (2024). AT-hook Motif Nuclear Localized Transcription Factors Function Redundantly in Promoting Root Growth through Modulation of Redox Homeostasis. Plant J..

[59] Lu M., Chen Z., Dang Y., Li J., Wang J., Zheng H., Li S., Wang X., Du X., Sui N. (2023). Identification of the MYB Gene Family in *Sorghum bicolor* and Functional Analysis of SbMYBAS1 in Response to Salt Stress. Plant Mol. Biol..

[60] Shukla P.S., Agarwal P., Gupta K., Agarwal P.K. (2015). Molecular Characterization of an MYB Transcription Factor from a Succulent Halophyte Involved in Stress Tolerance. AoB Plants.

[61] Li W., Li H., Wei Y., Han J., Wang Y., Li X., Zhang L., Han D. (2024). Overexpression of a *Fragaria vesca* NAM, ATAF, and CUC (NAC) Transcription Factor Gene (FvNAC29) Increases Salt and Cold Tolerance in *Arabidopsis thaliana*. Int. J. Mol. Sci..

[62] Zhang L., Xing L., Dai J., Li Z., Zhang A., Wang T., Liu W., Li X., Han D. (2024). Overexpression of a Grape WRKY Transcription Factor VhWRKY44 Improves the Resistance to Cold and Salt of *Arabidopsis thaliana*. Int. J. Mol. Sci..

[63] Liu X., Zhou G., Chen S., Jia Z., Zhang S., Ren M., He F. (2023). Genome-Wide Analysis of the AP2/ERF Gene Family in Tritipyrum and the Response of TtERF_B2-50 in Salt-Tolerance. BMC Genom..

[64] Wei Y., Li Z., Lv L., Yang Q., Cheng Z., Zhang J., Zhang W., Luan Y., Wu A., Li W. (2023). Overexpression of MbICE3 Increased the Tolerance to Cold and Drought in Lettuce (*Lactuca sativa* L.). Vitr. Cell. Dev. Biol.-Plant.

[65] Wang X., Li Y., Chen Z., Li L., Li Q., Geng Z., Liu W., Hou R., Zhang L., Han D. (2025). MbWRKY50 Confers Cold and Drought Tolerance through Upregulating Antioxidant Capacity Associated with ROS Scavenging. J. Plant Physiol..

[66] Su H., Cao L., Ren Z., Sun W., Zhu B., Ma S., Sun C., Zhang D., Liu Z., Zeng H. (2024). ZmELF6-ZmPRR37 Module Regulates Maize Flowering and Salt Response. Plant Biotechnol. J..

[67] Huo C., He L., Yu T., Ji X., Li R., Zhu S., Zhang F., Xie H., Liu W. (2022). The Superoxide Dismutase Gene Family in *Nicotiana tabacum*: Genome-Wide Identification, Characterization, Expression Profiling and Functional Analysis in Response to Heavy Metal Stress. Front. Plant Sci..

[68] Wang M., Ma Y., Qiu Y.-X., Long S.-S., Dai W.-S. (2025). Genome-Wide Characterization and Expression Profiling of the TGA Gene Family in Sweet Orange (*Citrus sinensis*) Reveal CsTGA7 Responses to Multiple Phytohormones and Abiotic Stresses. Front. Plant Sci..

[69] Zhan F., Wang Y., Zhang L., Yu Y., Ni Z. (2025). Genome-Wide Identification of the OVATE Gene Family of Proteins in Soybean and Expression Profiling under Salt Stress. Front. Plant Sci..

[70] Mas-ud M.A., Yin C., Juthee S.A., Hosenuzzaman M., Haque M.E., Zhu Y., Haque M.A., Matin M.N. (2025). Comprehensive Genome-Wide Identification and Analysis of MYB Transcription Factors Related to Abiotic and Biotic Stress Regulation in Rice. Sci. Rep..

[71] Zhang F., Ma J., Liu Y., Fang J., Wei S., Xie R., Han P., Zhao X., Bo S., Lu Z. (2024). A Multi-Omics Analysis Revealed the Diversity of the MYB Transcription Factor Family’s Evolution and Drought Resistance Pathways. Life.

[72] Yuan H., Cheng M., Wang R., Wang Z., Fan F., Wang W., Si F., Gao F., Li S. (2024). miR396b/*GRF6* Module Contributes to Salt Tolerance in Rice. Plant Biotechnol. J..

[73] Yoshida T., Mogami J., Yamaguchi-Shinozaki K. (2014). ABA-Dependent and ABA-Independent Signaling in Response to Osmotic Stress in Plants. Curr. Opin. Plant Biol..

[74] Hasanuzzaman M., Bhuyan M.H.M.B., Parvin K., Bhuiyan T.F., Anee T.I., Nahar K., Hossen M.S., Zulfiqar F., Alam M.M., Fujita M. (2020). Regulation of ROS Metabolism in Plants under Environmental Stress: A Review of Recent Experimental Evidence. Int. J. Mol. Sci..

[75] Goodstein D.M., Shu S., Howson R., Neupane R., Hayes R.D., Fazo J., Mitros T., Dirks W., Hellsten U., Putnam N. (2012). Phytozome: A Comparative Platform for Green Plant Genomics. Nucleic Acids Res..

[76] Jin J., Tian F., Yang D.-C., Meng Y.-Q., Kong L., Luo J., Gao G. (2017). PlantTFDB 4.0: Toward a Central Hub for Transcription Factors and Regulatory Interactions in Plants. Nucleic Acids Res..

[77] Camacho C., Coulouris G., Avagyan V., Ma N., Papadopoulos J., Bealer K., Madden T.L. (2009). BLAST+: Architecture and Applications. BMC Bioinform..

[78] Bateman A., Martin M.-J., Orchard S., Magrane M., Ahmad S., Alpi E., Bowler-Barnett E.H., Britto R., Bye-A-Jee H., The UniProt Consortium (2023). UniProt: The Universal Protein Knowledgebase in 2023. Nucleic Acids Res..

[79] Letunic I., Bork P. (2019). Interactive Tree of Life (iTOL) v4: Recent Updates and New Developments. Nucleic Acids Res..

[80] Duvaud S., Gabella C., Lisacek F., Stockinger H., Ioannidis V., Durinx C. (2021). Expasy, the Swiss Bioinformatics Resource Portal, as Designed by Its Users. Nucleic Acids Res..

[81] Lescot M. (2002). PlantCARE, a Database of Plant Cis-Acting Regulatory Elements and a Portal to Tools for in Silico Analysis of Promoter Sequences. Nucleic Acids Res..

[82] Shannon P., Markiel A., Ozier O., Baliga N.S., Wang J.T., Ramage D., Amin N., Schwikowski B., Ideker T. (2003). Cytoscape: A Software Environment for Integrated Models of Biomolecular Interaction Networks. Genome Res..

[83] Xu L., Zheng Y., Yu Q., Liu J., Yang Z., Chen Y. (2022). Transcriptome Analysis Reveals the Stress Tolerance to and Accumulation Mechanisms of Cadmium in *Paspalum vaginatum* Swartz. Plants.

[84] Zhang J., Zhang C., Huang S., Chang L., Li J., Tang H., Dey S., Biswas A., Du D., Li D. (2021). Key Cannabis Salt-Responsive Genes and Pathways Revealed by Comparative Transcriptome and Physiological Analyses of Contrasting Varieties. Agronomy.

[85] Love M.I., Huber W., Anders S. (2014). Moderated Estimation of Fold Change and Dispersion for RNA-Seq Data with DESeq2. Genome Biol..

[86] Zhang T., Cui Z., Li Y., Kang Y., Song X., Wang J., Zhou Y. (2021). Genome-Wide Identification and Expression Analysis of MYB Transcription Factor Superfamily in *Dendrobium catenatum*. Front. Genet..

[87] Huerta-Cepas J., Szklarczyk D., Heller D., Hernández-Plaza A., Forslund S.K., Cook H., Mende D.R., Letunic I., Rattei T., Jensen L.J. (2019). eggNOG 5.0: A Hierarchical, Functionally and Phylogenetically Annotated Orthology Resource Based on 5090 Organisms and 2502 Viruses. Nucleic Acids Res..

[88] Sun W., Ma Z., Chen H., Liu M. (2019). MYB Gene Family in Potato (*Solanum tuberosum* L.): Genome-Wide Identification of Hormone-Responsive Reveals Their Potential Functions in Growth and Development. Int. J. Mol. Sci..

[89] Wu X., Shi H., Guo Z. (2018). Overexpression of a NF-YC Gene Results in Enhanced Drought and Salt Tolerance in Transgenic Seashore Paspalum. Front. Plant Sci..

[90] Schmittgen T.D., Livak K.J. (2008). Analyzing Real-Time PCR Data by the Comparative CT Method. Nat. Protoc..

[91] Ke Y., Abbas F., Zhou Y., Yu R., Yue Y., Li X., Yu Y., Fan Y. (2019). Genome-Wide Analysis and Characterization of the Aux/IAA Family Genes Related to Floral Scent Formation in *Hedychium coronarium*. Int. J. Mol. Sci..

